# Cystargolide-based amide and ester Pz analogues as proteasome inhibitors and anti-cancer agents

**DOI:** 10.1098/rsos.220358

**Published:** 2022-09-28

**Authors:** Carlos R. Viera, Bradley T. Stevens, Talysa Viera, Cameron Zielinski, Lee A. Uranga, Snezna Rogelj, Praveen L. Patidar, Rodolfo Tello-Aburto

**Affiliations:** ^1^ Department of Chemistry, New Mexico Institute of Mining and Technology, Socorro, NM 87801, USA; ^2^ Department of Biology, New Mexico Institute of Mining and Technology, Socorro, NM 87801, USA; ^3^ Department of Chemical Engineering, New Mexico Institute of Mining and Technology, Socorro, NM 87801, USA; ^4^ Department of Chemistry and Biochemistry, New Mexico State University, Las Cruces, NM 88003, USA

**Keywords:** proteasome inhibitors, β-lactones, multiple myeloma

## Abstract

A series of cystargolide-based β-lactone analogues containing nitrogen atoms at the Pz portion of the scaffold were prepared and evaluated as proteasome inhibitors, and for their cytotoxicity profile toward several cancer cell lines. Inclusion of one, two or even three nitrogen atoms at the Pz portion of the cystargolide scaffold is well tolerated, producing analogues with low nanomolar proteasome inhibition activity, in many cases superior to carfilzomib. Additionally, analogue **8g**, containing an ester and pyrazine group at Pz, was shown to possess significant activity toward RPMI 8226 cells (IC_50_ = 21 nM) and to be less cytotoxic toward the normal tissue model MCF10A cells than carfilzomib.

## 1. Introduction

The ubiquitin–proteasome system (UPS) is the main mechanism of protein degradation in eukaryotic cells [1]. Since its discovery in 1977 by Goldberg & Etlinger [2], the function and regulation of the UPS have been associated with many important cellular homeostasis processes such as cell cycle progression, survival, and triggering of apoptosis [3]. Although the proper function of the UPS is critical for all cells, cancer cells can be more sensitive to inhibition of the UPS due to the increased proteolytic activity required during the high protein stress associated with uncontrolled cell division. As such, inhibition of the proteasome has been validated as a useful strategy for cancer therapy [4,5]. The boronic acid bortezomib is the first-in-class FDA-approved proteasome inhibitor (PI) for the treatment of multiple myeloma and mantle cell lymphoma [6]. Despite its utility, bortezomib has been associated with peripheral neuropathy in more than 30% of patients [7]. Other FDA-approved PIs include the orally available boronic acid derivative ixazomib, and the epoxyketone carfilzomib. Treatment with carfilzomib carries less risk of peripheral neuropathy than bortezomib [7]; however, carfilzomib has been associated with the development of cardiovascular conditions such hypertension and heart failure [8]. The safety profile of ixazomib is still under investigation, and at least in one case, it has been associated with the development of Sweet's syndrome [9].

**Figure 1.**
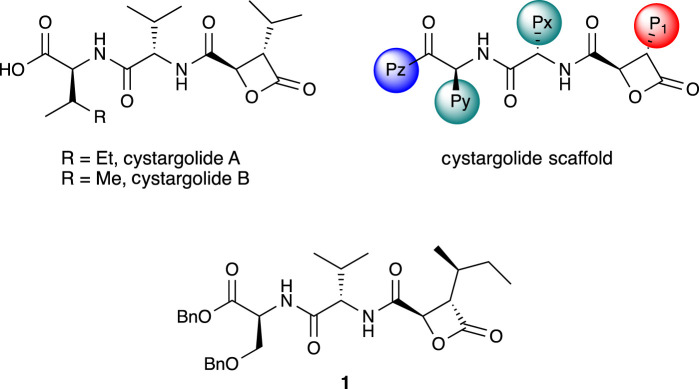
Cystargolides A and B, cystargolide scaffold and analogue **1**.

Natural products have been a traditionally rich source of biologically active compounds, including proteasome inhibitors [10]. Among these, the β-lactone-γ-lactams clasto-lactacystin-β-lactone [11,12] (omuralide) and the salinosporamides [13] have received considerable attention in recent years [5]. Other β-lactones such as the belactosins [14,15] and cystargolides [16] represent a promising class of naturally occurring proteasome inhibitors, yet they possess a simpler structure than the β-lactone-γ-lactams. The belactosins have been the subject of several synthetic [17–20] and optimization [21–25] studies. Cystargolides A and B were isolated from the actinomycete *Kitasatospora cystarginea* and were shown to inhibit the activity of the human proteasome at micromolar concentrations. Our group achieved the first total synthesis and elucidated the absolute stereochemistry of both cystargolides A and B [26]. The work also delivered two benzyl ester derivatives that showed improved proteasome inhibition activity, as well as promising cytotoxicity toward MCF-7 breast cancer cells. Based on our initial studies, a series of synthetic derivatives were prepared, focusing on three main structural diversification areas of the cystargolide scaffold: the β-lactone side chain (P_1_), the dipeptide core (Px and Py), and the end-cap (Pz) [27] (figure 1).

Evaluation of these first-generation analogues led to **1**, possessing an isobutyl group at P_1_, L-valine and O-benzyl L-serine residues at the dipeptide portion, and a benzyl ester at Pz (figure 1). Analogue **1** emerged as a potent inhibitor of the *β*5 subunit of human proteasomes (IC_50_ = 3.1 nM) which promotes significant cytotoxicity toward MCF-7 (IC_50_ = 416 nM), MDA-MB-231 (IC_50_ = 74 nM) and RPMI 8226 (IC_50_ = 41 nM) cancer cell lines. The efficacy of **1** toward multiple myeloma cells approaches that of carfilzomib; however, comparison of the proteasome inhibition in cell lysate and in whole cells indicated that cellular infiltration remained the primary barrier for further optimization of **1** and related PIs [27].

In order to improve the pharmacokinetic profiles of our proteasome inhibitors, we hypothesized that increasing the number of nitrogen atoms in the structure could deliver additional analogues with improved aqueous solubility and activity. According to our previous observations [27], the end-cap (Pz) portion of the cystargolide scaffold (figure 1) seemed to better tolerate structural changes while preserving desirable inhibitory activity and cytotoxicity profiles. For this reason, we synthesized and evaluated cystargolide-based Pz analogues incorporating ester or amide linkages, and pyridine or pyrazine aromatic moieties. The resulting nitrogenated analogues reported here hold nanomolar proteasome inhibition activity and show significant cytotoxity toward several cancer cell lines.

## 2. Chemistry

**Scheme 1.**
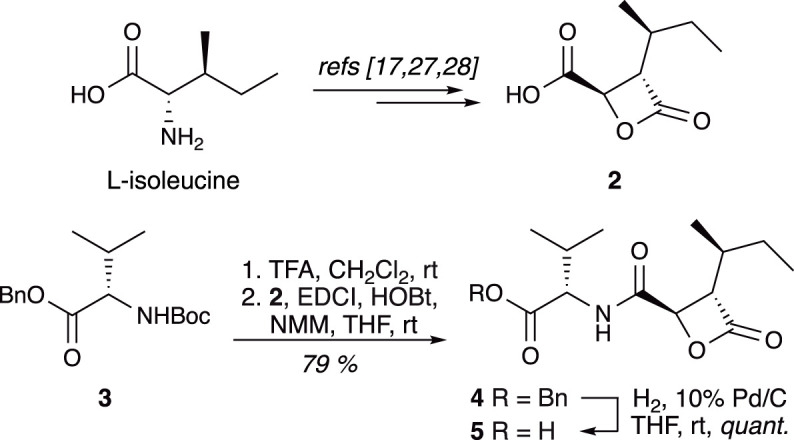
Synthesis of common intermediate **5**.

The construction of our nitrogenated Pz analogues was based on methodology used in our previous work on the synthesis of cystargolide derivatives [27]. Known β-lactone **2** [17,27,28] can be efficiently prepared from L-isoleucine by a sequence of reactions involving deamination, asymmetric alkylation and one-pot chlorination/cyclization (scheme 1). Coupling of **2** with the TFA salt derived from known N-Boc O-benzyl L-valine **3** [29] followed by hydrogenolysis yielded common intermediate **5** in good yield (scheme 1).

**Scheme 2.**
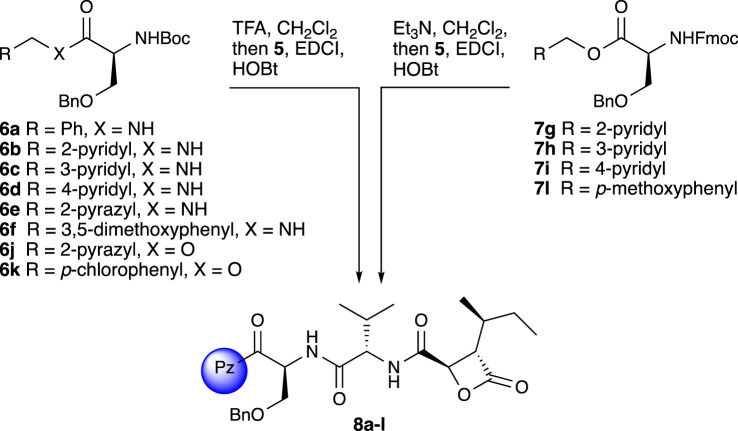
Synthesis of analogues **8a–l**.

Incorporation of the nitrogenated substituents at the Pz portion was then carried out by coupling of **5** with the free amine or salt derived from either N-Boc or N-Fmoc protected O-benzyl serine derivatives **6** and **7** (scheme 2).

Table 1 shows a summary of structures for the prepared analogues **8a–8l**. All new compounds gave satisfactory NMR and HRMS analyses. For example, ^1^H NMR spectra showed the expected signals for the protons of a *trans*-β-lactone moiety between *δ* 3.5 and 5.00 ppm as well as doublet and triplet methyl group signals between *δ*0.5 and 0.9 ppm for the β-lactone side chain. All ^13^C NMR spectra for the final products showed the appropriate number of signals with expected chemical shifts for the products, for example, displaying the corresponding lactone, ester and/or amide carbonyl signals between *δ* 165 and 175 ppm (see electronic supplementary material for details).

**Table 1.** Structures of amide and ester analogues.

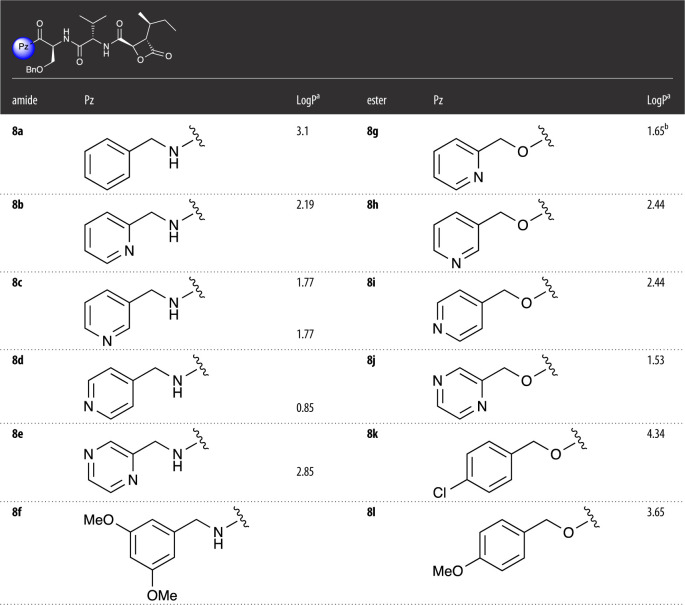

^a^Calculated using the chemical properties function of ChemDraw v. 21.0.

^b^Experimentally determined (see electronic supplementary material for details).

## 3. Proteasome inhibition

Based on our previously reported procedure [27], the degree of peptide cleavage in a crude Jurkat cell lysate was measured to determine proteasome activity. The fluorescent substrate Suc-LLVY-AMC (Enzo Life Sciences) was used to determine chymotrypsin-like (***β***5) activity of the proteasomes. The clinically used drug carfilzomib was included for comparison.

As shown in table 2, incorporation of nitrogen substitution at the end cap (Pz) produced analogues **8a–8j**, with potent proteasome inhibition activity. Every analogue containing at least one nitrogen atom at Pz, with the exception of **8j**, showed proteasome inhibition activity superior to that of carfilzomib (IC_50_ = 9.6 nM in the same assay). Non-nitrogenated Pz analogues **8k** and **8l**, which also contain a *para*-substitution at the Pz aromatic ring, were found to perform below the activity of carfilzomib in the same test. These results indicate that the replacement of an ester linkage by an amide at the Pz portion of our PIs is a viable option while preserving the increased proteasome inhibition of our analogues with respect to carfilzomib. Besides the fact that amides are generally more polar than comparable esters, and hence potentially more water soluble, the incorporation of an amide linkage at the Pz portion of the scaffold might also be relevant to increase the chemical and metabolic stability of our PIs (the half-life of **8g** in buffer was found to be greater than 10 min and the biological stability greater than 5 min in serum; see electronic supplementary material). Isosteric replacement of the phenyl ring at Pz by either a pyridyl or pyrazyl group was well tolerated, allowing the inclusion of one (compounds **8b–d** and **8g–i**) or two nitrogen atoms (compounds **8e** and **8j**).

**Table 2.** Proteasome inhibition.

proteasome inhibition IC_50_ (µM) ± s.d.					
compound	Pz	h*β*5	compound	Pz	h*β*5
**1**	BnO	0.0028 ± 0.0003	**8g**	*2-Pyr-*CH_2_-*O*	0.0071 ± 0.0016
**8a**	BnNH	0.0023 ± 0.0009	**8h**	*3-Pyr-*CH_2_-O	0.0033 ± 0.0018
**8b**	*2-Pyr-*CH_2_-NH	0.0041 ± 0.0019	**8i**	*4-Pyr-*CH_2_-O	0.0040 ± 0.0011
**8c**	*3-Pyr-*CH_2_-NH	0.0039 ± 0.0018	**8j**	*2-Pyraz-*CH_2_-O	0.0137 ± 0.0016
**8d**	*4-Pyr-*CH_2_-NH	0.0030 ± 0.0012	**8k**	*p-Cl-BnO*	0.0182 ± 0.054
**8e**	*2-Pyraz-*CH_2_-NH	0.0084 ± 0.0034	**8l**	*p-MeO-BnO*	0.0188 ± 0.0050
**8f**	*3,5-DiMeO-Bn-NH*	0.0045 ± 0.0015	**carfilzomib**		0.0096 ± 0.0006

## 4. Docking studies

**Figure 2.**
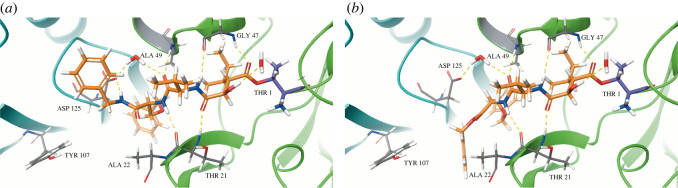
(*a*) Docking of amide **8a** (orange) and (*b*) docking of benzyl ester 1 (orange) with the *β*5 subunit of the proteasome (green). Thr1 residue is shown in purple, with the adjacent *β*6 subunit of the proteasome in teal.

The replacement of an ester by an amide at the end-cap linkage of our analogues also introduces an additional hydrogen bond donor at this position. We hypothesized that this additional hydrogen bond donor could provide additional stabilization in the binding pocket of the proteasome. To test this idea, we used CovDock [30] within the Schrodinger docking suite Maestro and conducted docking studies of amide **8a** with the *β*5 subunit of the human 20S proteasome. The X-ray crystal structure of the 20S proteasome bound to carfilzomib (PDB: 4R67) was used to define the binding pocket where our PIs could accommodate upon entry to the catalytic site. After performing non-covalent ligand docking, we observed that the β-lactone carbonyl of **8a** placed approximately 3.6 Å away from the nucleophilic hydroxyl group at the proteasome Thr1 residue (figure 2). Covalent docking was then implemented to show that after acyl-ester formation, **8a** is stabilized by a network of hydrogen bond interactions between its peptidic backbone and residues Thr21, Ala22, Gly47 and Ala49, as well as nearby water molecules (figure 2). These interactions are also observed for benzyl ester **1** (figure 2); however, amide **8a** takes advantage of an additional hydrogen bonding interaction with an Asp125 residue at the nearby *β*6 subunit, which is not present for benzyl ester **1**.

## 5. Cytotoxicity studies

Next, analogues **8a–l** were evaluated for their cytotoxicities toward Jurkat cells, a human T-cell leukemia model, as well as the breast cancer cell lines MCF-7 and MDA-MB-231, and the multiple myeloma cell line RPMI 8226. A summary of the observed IC_50_ values for each compound and cell line is shown in table 3. Our previously described analogue **1** and the commercial drug carfilzomib were included for comparison.

**Table 3.** Cytotoxicity evaluation.

cytotoxicity IC_50_ (µM) ± s.d.					
compound	Pz	Jurkat	RPMI 8226	MCF-7	MDA-MB-231
**1**	BnO	0.062 ± 0.007	0.045 ± 0.0005	2.376 ± 0.261	0.620 ± 0.050
**8a**	BnNH	0.012 ± 0.002	0.064 ± 0.004	2.293 ± 0.195	1.941 ± 0.166
**8b**	*2-Pyr-*CH_2_-NH	0.058 ± 0.014	0.139 ± 0.006	0.453 ± 0.024	0.156 ± 0.003
**8c**	*3-Pyr-*CH_2_-NH	0.071 ± 0.019	0.085 ± 0.003	5.629 ± 0.550	0.242 ± 0.006
**8d**	*4-Pyr-*CH_2_-NH	0.064 ± 0.010	0.037 ± 0.001	5.298 ± 0.598	0.328 ± 0.012
**8e**	*2-Pyraz-*CH_2_-NH	0.132 ± 0.004	0.031 ± 0.002	5.177 ± 0.602	0.255 ± 0.006
**8f**	*3,5-DiMeO-BnNH*	0.063 ± 0.002	0.026 ± 0.001	0.322 ± 0.039	0.053 ± 0.002
**8g**	*2-Pyr-*CH_2_-*O*	0.035 ± 0.010	0.021 ± 0.003	1.019 ± 0.042	0.039 ± 0.002
**8h**	*3-Pyr-*CH_2_-O	0.061 ± 0.016	0.043 ± 0.002	3.799 ± 0.293	0.132 ± 0.003
**8i**	*4-Pyr-*CH_2_-O	0.036 ± 0.010	0.035 ± 0.003	4.927 ± 0.403	0.108 ± 0.007
**8j**	*2-Pyraz-*CH_2_-O	0.166 ± 0.009	0.048 ± 0.002	4.531 ± 0.290	0.146 ± 0.003
**8k**	*p-Cl-BnO*	0.182 ± 0.015	0.182 ± 0.004	3.501 ± 0.279	0.222 ± 0.015
**8l**	*p-MeO-BnO*	2.260 ± 0.158	0.112 ± 0.002	1.670 ± 0.267	0.636 ± 0.047
**carfilzomib**		0.0037 ± 0.0003	0.0037 ± 0.0003	0.0059 ± 0.0007	0.0044 ± 0.0001

As expected, carfilzomib showed potent activity across all cell lines tested, especially toward the blood cancer cells Jurkat and RPMI 8226, where it exhibited single-digit nanomolar IC_50_ values. Benzyl ester **1** and nitrogenated analogues **8a–8j** also showed significant activity toward Jurkat cells, with IC_50_ values ranging from 12 to 166 nM. Analogues **8k** and **8l** containing a *para*-substitution at the Pz aromatic ring were in general less potent. Comparing the activity of our PIs toward the two breast cancer cell lines tested, we observed that all of our analogues show a preference for killing triple-negative MDA-MB-231 cells over estrogen-receptor-positive MCF-7 cells.

From this entire study, compound **8g**, possessing a 2-pyridyl ester at Pz, emerged as the best analogue, showing potent cytotoxicity toward Jurkat cells (IC_50_ = 35 nM), MDA-MB-231 cells (IC_50_ = 39 nM) and RPMI 8226 cells (IC_50_ = 21 nM), Notably, **8g** is more than twice as potent toward RPMI 8226 cells than our previously described lead **1** (IC_50_ = 45 nM in the same assay). Evaluation of **8g** toward MCF-10A cells as a model for normal breast tissue resulted in an IC_50_ value of 123 nM, comparing favourably to carfilzomib, which showed an IC_50_ value of 79 nM in the same assay.

In summary, a series of cystargolide-based Pz analogues were prepared and evaluated for their ability to inhibit the activity of the 20S proteasome, as well as their cytotoxicity. The activity profile observed for nitrogen-containing analogues **8a–8j** demonstrated that addition of either one, two or even three nitrogen atoms at the Pz portion of the cystargolide scaffold is well tolerated, and in most cases delivered proteasome inhibitors that are more potent than the commercially used drug carfilzomib. Cytotoxicity evaluation showed a preference of our analogues for killing blood cancer cells versus breast cancer cells and delivered compound **8g** as the best analogue of this entire study. Compound **8g** possesses a 2-pyridyl moiety at the Pz portion, and shows significant activity toward Jurkat, MCF-7, MDA-MB-231 and RPMI cells. Compound **8g** also showed less cytotoxicity toward the normal tissue model MCF10A cells than carfilzomib. The incorporation of nitrogen atoms at the Pz portion of the cystargolide scaffold is a viable strategy that might contribute to improving the solubility and stability of our analogues, which in turn will be relevant for the design of further optimized proteasome inhibitors with desirable anti-cancer activity and improved pharmacokinetic profiles. Efforts toward further optimization of our analogues are currently ongoing in our laboratories, and the results from these investigations will be reported in due course.

## Data Availability

Our data have been deposited at Dryad Digital Repository: https://doi.org/10.5061/dryad.hmgqnk9kk [31]. The data are provided in the electronic supplementary material [32].
